# Endoscopic submucosal dissection of a tumor in the upper esophageal sphincter and piriform sinus

**DOI:** 10.1016/j.vgie.2022.08.009

**Published:** 2022-09-27

**Authors:** Raymond Kim, Ahmed Chatila

**Affiliations:** Department of Gastroenterology and Hepatology, University of Maryland, Baltimore, Maryland

**Keywords:** ESD, endoscopic submucosal dissection, NBI, narrow band imaging, PET, positron emission tomography, SCC, squamous cell carcinoma

## Abstract

Video 1 Endoscopic submucosal dissection of a tumor in the upper esophageal sphincter and piriform sinus.

Endoscopic submucosal dissection of a tumor in the upper esophageal sphincter and piriform sinus.

We present a case of a 77-year-old man who underwent endoscopic submucosal dissection of a tumor in the upper esophageal sphincter and piriform sinus for a poorly differentiated squamous cell carcinoma. The patient presented initially for a routine EGD for GERD, which showed mucosal irregularity at the upper esophageal sphincter and piriform sinus with a biopsy showing a squamous cell carcinoma. A positron emission tomography (PET) CT scan done shortly after showed no focal uptake or lymphadenopathy. He had a subsequent EUS (Fig. 1) with a biopsy 1 month later, again revealing a raised mass at the upper esophageal sphincter with pathology demonstrating a poorly differentiated squamous cell carcinoma (SCC) (uT1a N0 MX). The case was reviewed at Tumor Board and, given surgical intervention was limited and would result in a potential laryngopharyngectomy and proximal esophagectomy, an endoscopic submucosal dissection with concurrent chemoradiation was decided to be pursued.

**Figure 1:**
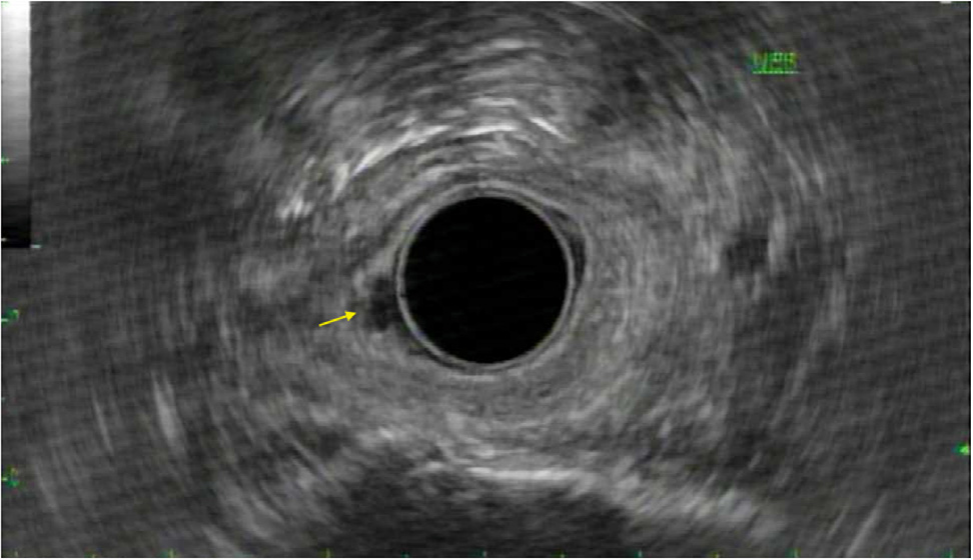
EUS of the endoscopic tumor in the upper esophageal sphincter and piriform sinus.

Prior to the dissection, the mass was visualized by repeating EUS and using both Lugol's solution and narrow-band imaging (NBI) to better assess the margins. During the endoscopic submucosal dissection (ESD), general sedation was achieved with Sevoflurane gas, used to relax smooth muscles and allow for airway protection. The borders of the lesion were marked using NBI (Figs. 2 and 3), followed by injection of Orise gel, which provided adequate lift of the lesion from the muscularis propria allowing for submucosal dissection to be carried out. A mucosotomy using a dual knife was used to make a circumferential incision around the lesion into the submucosa (Fig. 4). The lesion was dissected from the underlying submucosal layers using a triangle knife with spray coagulation at 50 watts to prevent bleeding in a highly vascular area (Fig. 5). A total of 25 mm of area was resected (Figs. 6 and 7), with pathology showing a pT1b SM1 poorly differentiated squamous cell carcinoma with negative deep and lateral margins, R0 resection, along with no lymphovascular invasion (Fig. 8). There were no adverse events noted during the procedure and no antibiotics were administered during or after the procedure. Following completion of the procedure, the patient completed adjuvant chemoradiation.

**Figure 2:**
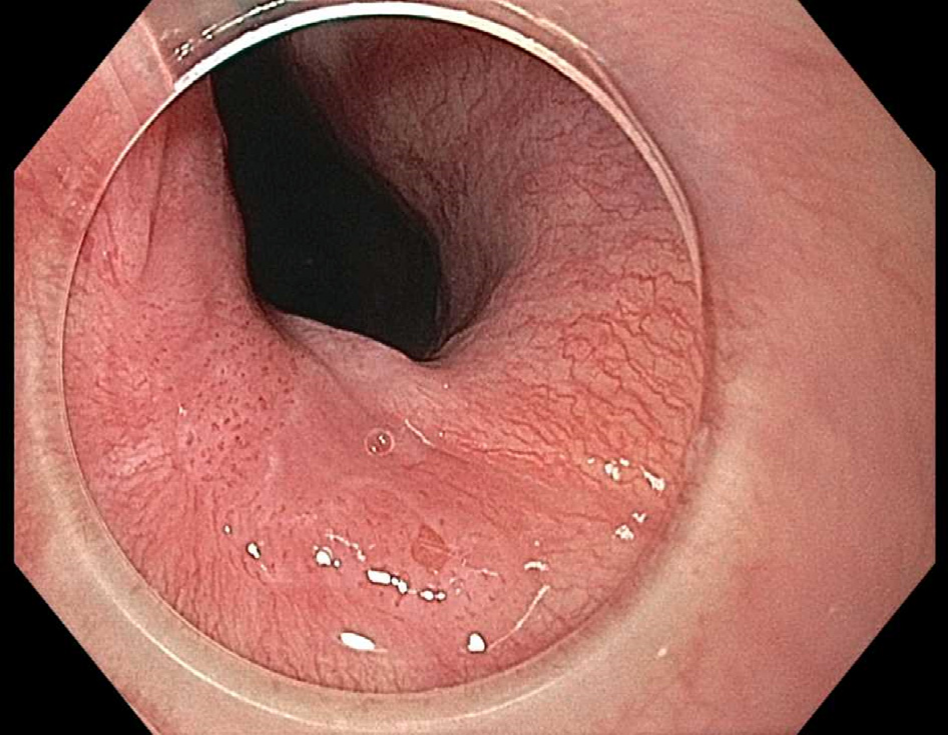
Tumor in the upper esophageal sphincter and piriform sinus.

**Figure 3:**
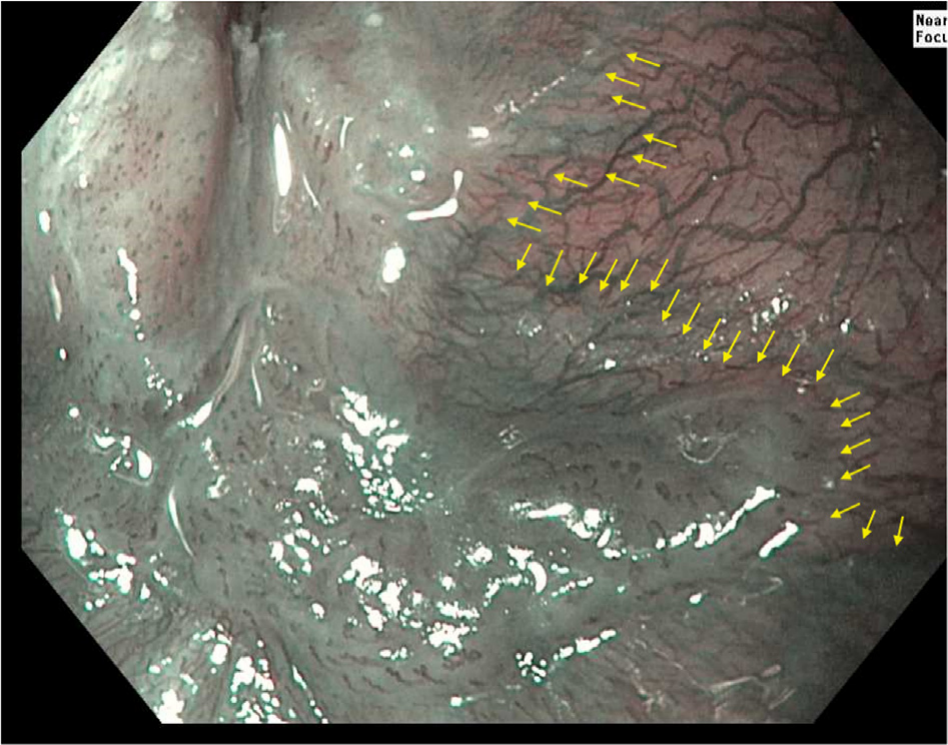
Tumor in the upper esophageal sphincter and piriform sinus under narrow-band imaging.

**Figure 4:**
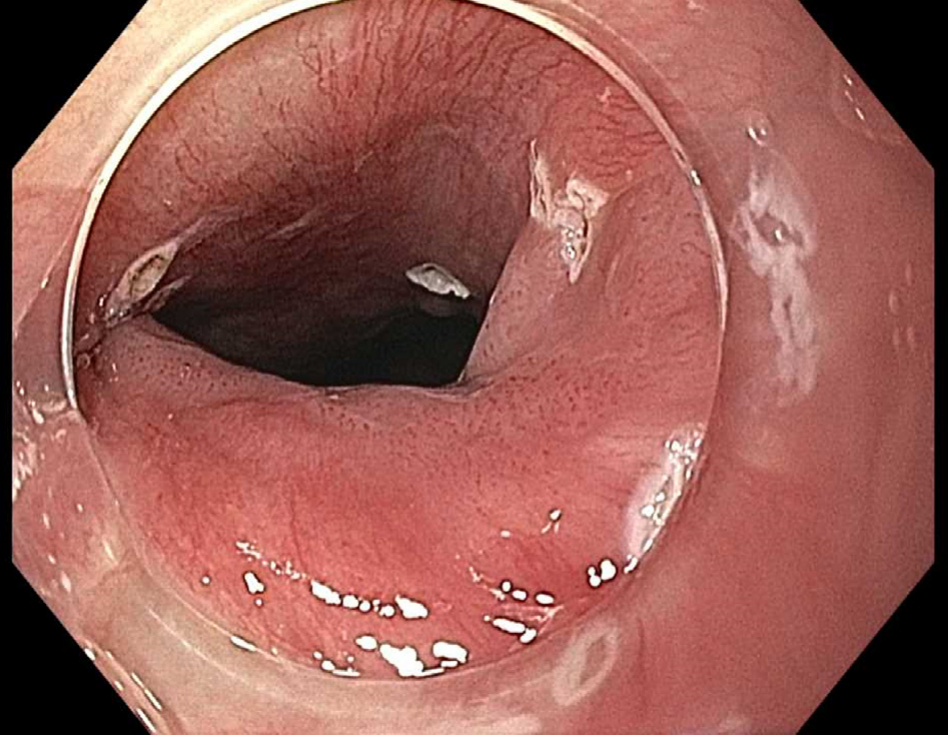
Demarcation of the endoscopic tumor.

**Figure 5:**
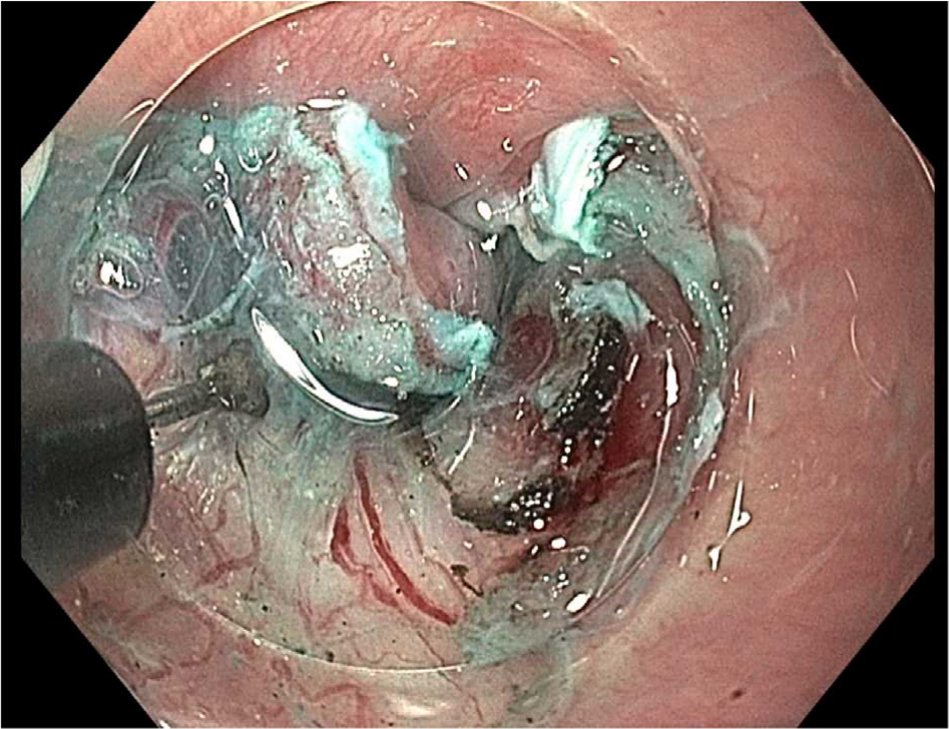
Tumor dissected from the underlying submucosal layers using a triangle knife.

**Figure 6:**
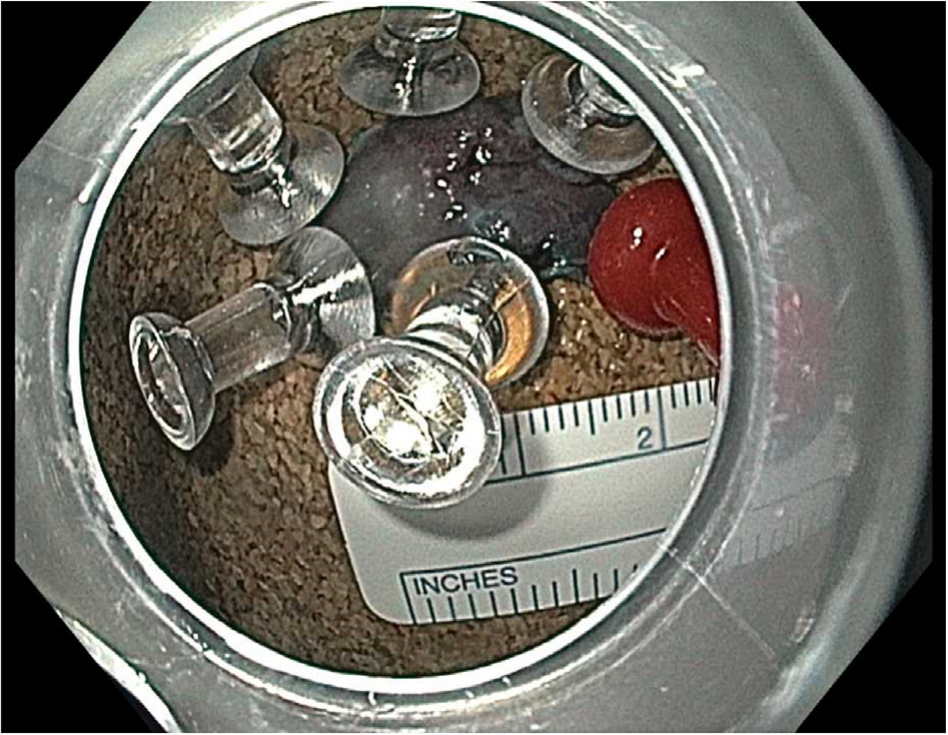
Resected tumor from the upper esophageal sphincter and piriform sinus.

**Figure 7:**
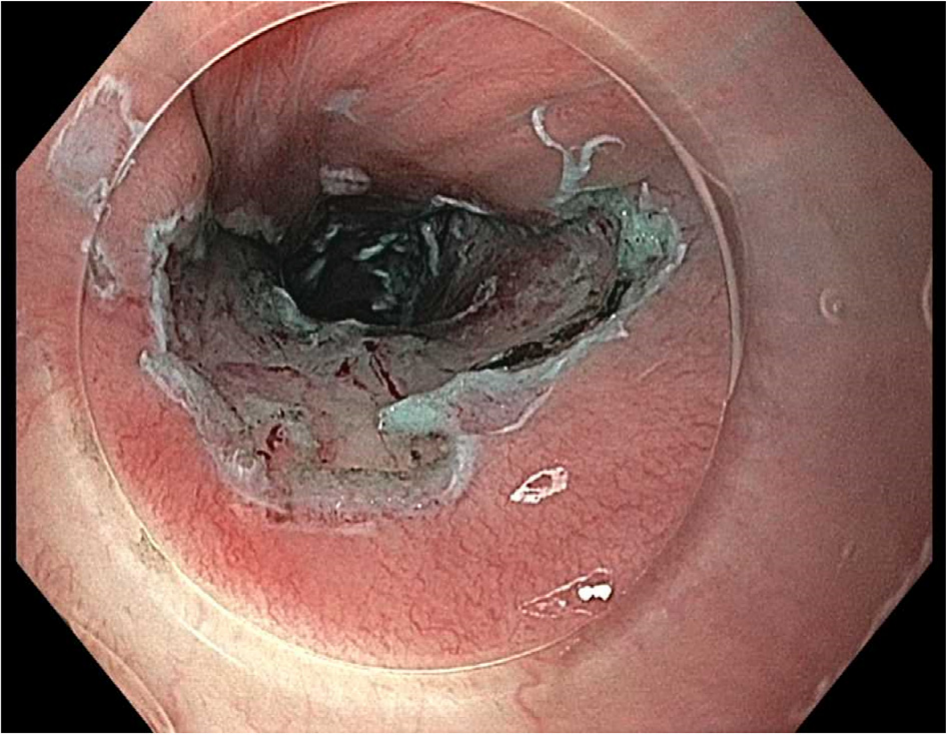
Resected area of the tumor in the upper esophageal sphincter and piriform sinus.

**Figure 8:**
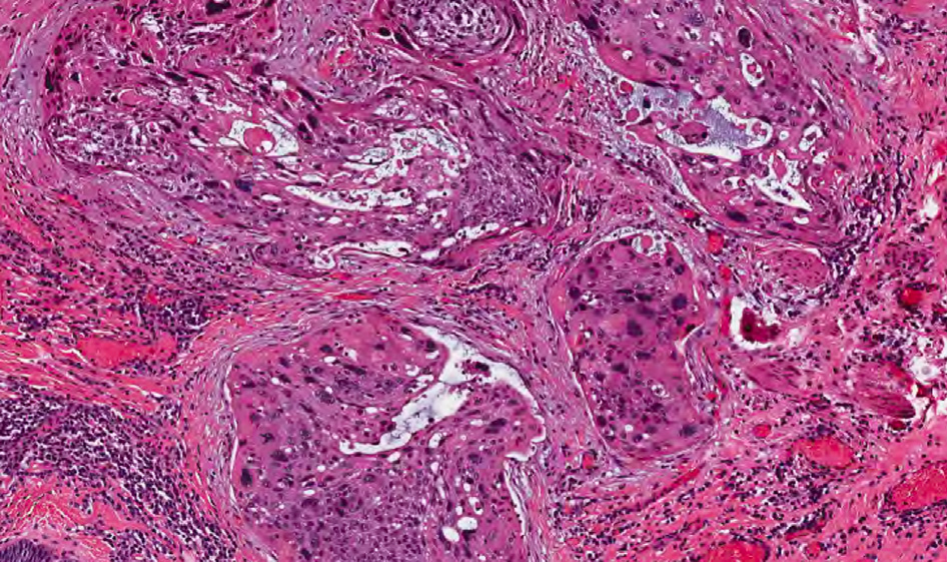
Pathology showing a pT1b SM1 poorly differentiated squamous cell carcinoma (H&E, orig. mag. ×40).

Six months after presentation, the patient began complaining of dysphagia with EGD showing stenosis at the resection site requiring dilation. He has since required repeat dilations for ongoing stenosis, though this has not affected his oral intake and no supplemental nutrition has been required. A PET CT scan done 10 months after presentation demonstrated focal metabolic uptake in the proximal right esophagus with a repeat EGD and EUS that month showing no mass or lymphadenopathy and repeat biopsies negative for recurrent disease. A repeat PET scan 13 months after presentation showed no evidence of recurrent or metastatic disease.

Endoscopic submucosal dissection has become a promising minimally invasive technique for resection of esophageal SCC. A recent meta-analysis demonstrated an overall 3-year survival of 90.5% and a 5-year survival of 87.3% following ESD of esophageal SCC.^1^ In particular, a study of ESDs for proximal esophageal SCCs showed similar survival rates to ESDs for esophageal SCC, with strictures noted in 16.7% of patients.^2^ Multiple methods have been used in the prevention of strictures after ESD, including intralesional injection of steroids, systemic steroids, injection of drugs targeting fibrotic changes (mitomycin C and N-acetylcysteine), and the use of transplanted materials and tissues to repair damaged tissue.^3^

Once again, this case suggests that resection of lesions in the cricopharyngeus and the upper esophageal sphincter and piriform sinus may circumvent the need for complex surgeries that would otherwise involve removal of the larynx and esophagus (Video 1, available online at www.giejournal.org).

## Disclosure

*Dr Kim is a consultant for Cook Medical and Medtronic. Dr Chatila disclosed no financial relationships. Informed consent was obtained for this article.*

## Supplementary data

Video 1 Endoscopic submucosal dissection of a tumor in the upper esophageal sphincter and piriform sinus.
